# Circular RNAs in cervical cancer: from ceRNA networks to epitranscriptomic regulation, immune modulation, and metastatic reprogramming

**DOI:** 10.3389/fimmu.2026.1848357

**Published:** 2026-07-24

**Authors:** Heying Huang, Lifeng Liu, Yuemei Cui, Fanchen Zhou, Jing Liu, Qianying Chen, Zhengyan Li, Bing Liu

**Affiliations:** 1 Department of Obstetrics and Gynaecology, Hospital of Chengdu University of Traditional Chinese Medicine, Chengdu, China; 2 Department of Obstetrics and Gynaecology, Central Hospital of Dalian University of Technology, Dalian, China

**Keywords:** cervical cancer, circular RNAs, epitranscriptomic regulation, immune modulation, metastatic reprogramming

## Abstract

Cervical cancer progression is driven not only by persistent high-risk human papillomavirus infection but also by multilayered post-transcriptional regulatory networks that reshape tumor cell behavior and the tumor microenvironment. Among these regulators, circular RNAs (circRNAs) have emerged as pivotal modulators of oncogenic signaling. Initially characterized as competing endogenous RNAs (ceRNAs), circRNAs were shown to promote cervical cancer growth, invasion, and chemoresistance by derepressing key oncogenic targets. However, recent evidence expands this paradigm, revealing that circRNAs are subject to epitranscriptomic modification and function as dynamic scaffolds integrating RNA-binding proteins, translational machinery, inflammatory signaling, and metabolic pathways. In cervical cancer, m6A-dependent regulation and reader-mediated translational control enhance circRNA stability and amplify oncogenic outputs, linking RNA modification to metabolic reprogramming and hypoxia adaptation. Concurrently, circRNAs modulate inflammatory cascades such as IL6/JAK/STAT3 and NF-κB, contributing to immune suppression and tumor microenvironment remodeling. These tumor-intrinsic and immune-extrinsic mechanisms converge on metastatic reprogramming, enabling lipid metabolic flexibility, lymphangiogenesis, autophagy activation, and therapeutic resistance. This review synthesizes current evidence to propose a unified regulatory landscape in which circRNAs function as central nodes connecting ceRNA circuits, epitranscriptomic modulation, immune signaling, and metabolic plasticity. Unlike previous reviews that primarily summarized circRNA-mediated ceRNA networks, canonical oncogenic pathways, or biomarker potential, this review adopts a systems-level perspective and critically integrates epitranscriptomic regulation, RNA-binding protein interactions, immune-inflammatory signaling, metabolic plasticity, and metastatic reprogramming. We further distinguish directly validated cervical cancer mechanisms from emerging or hypothetical regulatory layers, thereby providing a clearer conceptual framework for future mechanistic and translational studies.

## Highlights

- circRNAs extend beyond ceRNA networks to epitranscriptomic and translational control.
- CircRNAs integrate inflammatory signaling, immune suppression, and metabolic rewiring.
- Targeting circRNA–m6A–immune axes offers new therapeutic opportunities in cervical cancer.

## 1 Introduction

Cervical cancer remains one of the most prevalent gynecological malignancies worldwide and continues to pose a substantial public health burden, particularly in low- and middle-income regions. Despite the implementation of prophylactic human papillomavirus (HPV) vaccination and screening programs, cervical cancer remains a leading cause of cancer-related mortality among women globally. Persistent infection with high-risk HPV subtypes, particularly HPV16 and HPV18, represents the principal etiological factor driving cervical carcinogenesis (1–5). Viral oncoproteins E6 and E7 promote malignant transformation through inactivation of p53 and RB tumor suppressor pathways, thereby inducing genomic instability and uncontrolled proliferation (6–10). While early-stage cervical cancer is often amenable to surgical resection or radiotherapy, advanced and metastatic disease remains difficult to cure (11–15). Lymph node metastasis, local recurrence, and distant dissemination significantly worsen prognosis (16–20). Moreover, resistance to platinum-based chemotherapy and emerging immunotherapeutic strategies continues to limit long-term survival. Increasing evidence suggests that cervical cancer progression is not solely governed by canonical genetic alterations but is also driven by dynamic post-transcriptional regulatory networks, metabolic adaptation, and tumor microenvironmental remodeling. Therefore, uncovering novel regulatory layers that contribute to tumor plasticity, immune escape, and metastatic evolution is critical for improving therapeutic outcomes.

Circular RNAs (circRNAs) are a class of endogenous, covalently closed RNA molecules generated through a noncanonical back-splicing process, in which a downstream splice donor is joined to an upstream splice acceptor of precursor mRNAs (21, 22). According to their genomic origin and structural composition, circRNAs can be broadly classified into exonic circRNAs, intronic circRNAs, and exon–intron circRNAs (23). Exonic circRNAs are predominantly localized in the cytoplasm and are frequently involved in post-transcriptional regulation, whereas intronic and exon–intron circRNAs may remain in the nucleus and participate in transcriptional or splicing regulation (24, 25). Unlike linear RNAs, circRNAs lack 5′ caps and 3′ poly(A) tails, which confers remarkable resistance to exonuclease-mediated degradation and allows them to remain relatively stable in cells, tissues, plasma, serum, and exosomes. Their high stability, tissue- and disease-specific expression patterns, and detectability in bodily fluids make circRNAs attractive candidates as functional regulators and potential noninvasive biomarkers in cancer (26, 27). The discovery and characterization of circRNAs have been facilitated by advances in high-throughput RNA sequencing, RNase R enrichment, bioinformatic identification of back-splice junctions, and experimental validation using divergent primers, quantitative reverse-transcription PCR, Sanger sequencing, fluorescence *in situ* hybridization, and loss- or gain-of-function assays. These approaches have revealed that circRNAs are not transcriptional noise, but rather conserved and regulated RNA species with context-dependent biological functions (28–30). In cancer biology, circRNAs may regulate gene expression at multiple levels, including miRNA sequestration, interaction with RNA-binding proteins (RBPs), modulation of parental gene transcription, regulation of mRNA stability, control of translation, protein scaffolding, and, in some cases, encoding functional peptides (31). Therefore, circRNAs have gradually emerged as important components of post-transcriptional and epitranscriptomic regulatory networks.

Early studies predominantly characterized circRNAs as competing endogenous RNAs (ceRNAs) that act as microRNA sponges. By sequestering tumor-suppressive miRNAs, circRNAs can relieve repression of oncogenic target genes, thereby promoting tumor growth, invasion, metastasis, and chemoresistance (32–34). In cervical cancer, numerous circRNA–miRNA–mRNA regulatory axes have been identified, linking circRNAs to key oncogenic drivers such as FOXM1, AKT1, CDK2, and VEGFA. These findings established the foundational paradigm that circRNAs participate in cervical cancer progression through post-transcriptional derepression of oncogenes (35, 36). Given the central role of persistent high-risk human papillomavirus infection, viral oncoprotein-driven transformation, and tumor microenvironmental remodeling in cervical carcinogenesis, circRNAs may provide an additional regulatory layer that connects oncogenic signaling, immune escape, metabolic adaptation, and therapeutic resistance (37–39). However, the biological functions of circRNAs extend far beyond the classical ceRNA model. Emerging evidence indicates that circRNAs can interact with RBPs, modulate mRNA stability and translation, scaffold protein complexes, regulate signaling pathway activation, and even encode functional peptides under specific conditions. Furthermore, circRNAs are subject to epitranscriptomic modifications, such as N6-methyladenosine (m6A) and N4-acetylcytidine (ac4C), which influence their stability, localization, turnover, and translational potential (40, 41). These modifications allow circRNAs to respond dynamically to cellular stress, hypoxia, inflammatory stimulation, and metabolic rewiring. In cervical cancer, such higher-order regulatory mechanisms are increasingly recognized as important contributors to tumor progression, immune modulation, metastatic plasticity, and treatment resistance. These discoveries have expanded the conceptual framework of circRNA biology from passive miRNA sponges to active regulators of gene expression, cellular signaling, and tumor ecosystem remodeling.

Although substantial progress has been made in identifying circRNAs involved in cervical cancer, most early studies were confined to isolated ceRNA axes. Such a reductionist perspective may underestimate the broader regulatory influence of circRNAs within oncogenic networks. In recent years, a new wave of research has revealed that circRNAs participate in multilayered regulatory systems encompassing epitranscriptomic modification, RNA-binding protein interactions, immune signaling modulation, and metabolic reprogramming. Specifically, m6A modification and its associated writers, erasers, and readers have been shown to regulate circRNA stability and translational activity, thereby integrating circRNAs into epigenetic and translational control circuits (42–46). Concurrently, circRNA–RBP interactions have emerged as critical mechanisms shaping mRNA fate and protein synthesis. Beyond tumor-intrinsic effects, circRNAs also influence inflammatory signaling pathways such as IL6/JAK/STAT3 and NF-κB, contributing to immune modulation and tumor microenvironment remodeling. Moreover, recent evidence connects circRNAs to hypoxia adaptation, lipid metabolism, autophagy, and lymphangiogenesis—processes central to metastatic reprogramming and tumor plasticity. Given these advances, there is a pressing need to move beyond the traditional ceRNA paradigm and adopt an integrated regulatory framework that captures the multifaceted roles of circRNAs in cervical cancer biology. In this review, we synthesize current evidence to delineate how circRNAs orchestrate cervical cancer progression through interconnected mechanisms spanning ceRNA networks, epitranscriptomic regulation, immune modulation, and metastatic reprogramming. By constructing a unified conceptual model, we aim to highlight emerging therapeutic opportunities and identify future research directions in circRNA-mediated oncogenic regulation.

Several recent reviews have comprehensively summarized the roles of circRNAs in cervical cancer. Yin et al. discussed the emerging biological and clinical significance of circRNAs in cervical cancer, with emphasis on their functions in tumor proliferation, invasion, metastasis, angiogenesis, chemoresistance, and biomarker potential (47). Matos et al. provided a systematic review of circRNA-related evidence in cervical cancer pathogenesis, highlighting circRNA–miRNA–mRNA regulatory axes and their association with malignant phenotypes (48). Heydarnia et al. further summarized circRNA-mediated regulation of key signaling pathways involved in HPV-related cervical neoplasms, including PI3K/Akt/mTOR, MAPK, EMT-related programs, and therapeutic implications (49). These reviews have established an important foundation for understanding circRNAs as regulatory molecules in cervical cancer. However, most available reviews remain largely organized around individual circRNA–miRNA–mRNA axes, canonical oncogenic pathways, or diagnostic and therapeutic potential. In contrast, the present review aims to provide a conceptually distinct and systems-oriented framework. Rather than reiterating circRNAs primarily as competing endogenous RNAs, we focus on how circRNAs function as multilayered regulatory nodes that integrate epitranscriptomic modification, RNA-binding protein interactions, translational control, inflammatory signaling, immune modulation, metabolic plasticity, and metastatic reprogramming. We further distinguish mechanisms directly validated in cervical cancer from emerging or still speculative regulatory layers, such as YTH-domain reader-mediated circRNA translation, exosomal circRNA-mediated immune communication, and circRNA-encoded micropeptides. Therefore, the added value of this review lies in reframing cervical cancer-associated circRNAs from isolated ceRNA regulators into dynamic components of tumor–immune–metabolic regulatory networks.

## 2 Classical ceRNA networks driving cervical cancer progression

The earliest and most extensively characterized functions of circular RNAs (circRNAs) in cervical cancer were framed within the competing endogenous RNA (ceRNA) model. In this paradigm, circRNAs act as molecular sponges that sequester tumor-suppressive microRNAs (miRNAs), thereby relieving repression of oncogenic mRNA targets (50–54). Although initially perceived as isolated regulatory axes, accumulating evidence indicates that these ceRNA circuits converge on fundamental oncogenic programs governing cell-cycle progression, PI3K–AKT signaling, epithelial–mesenchymal transition (EMT), angiogenesis, and chemoresistance (55–59). Collectively, classical ceRNA networks constitute the foundational regulatory layer through which circRNAs contribute to cervical cancer progression. To provide an early integrative framework for this section, **Table 1** summarizes representative circRNA–miRNA–mRNA axes in cervical cancer and categorizes them according to their dominant biological outputs, including proliferation, migration and invasion, angiogenesis, autophagy, and chemoresistance.

**Table 1:** Classical ceRNA networks driving cervical cancer progression.

circRNA	miRNA target	Downstream effector	Functional outcome	Key biological process
circCLK3	miR-320a	FOXM1	Increased proliferation	Cell cycle progression
circ_0000520	miR-1296	CDK2	G1/S transition	Cell cycle activation
circRNA-AKT1	miR-942-5p	AKT1	Enhanced survival & growth	PI3K–AKT signaling
circZFR	—	CDK2/Cyclin E1	RB phosphorylation	Proliferation
circ_0023404	miR-5047	VEGFA	Angiogenesis & invasion	EMT & vascular remodeling
circ0001955	miR-188-3p	NCAPG2	Increased migration	Cytoskeletal remodeling
circ_0005576	miR-153	KIF20A	Enhanced motility	EMT
circMTO1	—	—	Chemoresistance	Survival adaptation
circPRDM4	—	Immune-related pathways	Immune escape	Therapy resistance

### 2.1 Proliferation-related axes

Uncontrolled proliferation is a hallmark of cervical carcinogenesis, and multiple circRNA-mediated ceRNA axes have been shown to directly modulate cell-cycle regulators and survival pathways.

One of the earliest identified oncogenic circuits involves circCLK3, which functions as a sponge for miR-320a, leading to derepression of the transcription factor FoxM1 (60). FOXM1 is a master regulator of cell-cycle progression, promoting transcription of genes required for G1/S and G2/M transitions. By sustaining FOXM1 expression, circCLK3 enhances proliferative capacity and accelerates tumor growth *in vitro* and *in vivo*. This axis exemplifies how circRNAs can influence transcriptional networks through post-transcriptional miRNA sequestration. Similarly, circ_0000520 promotes cervical cancer cell proliferation by targeting miR-1296 and upregulating CDK2, a central driver of G1/S transition (61). Increased CDK2 activity facilitates cyclin E–dependent phosphorylation events, pushing cells through the restriction point and promoting DNA replication. In parallel, circZFR has been shown to enhance phosphorylation of the RB protein through regulation of the CDK2/cyclin E1 complex, further reinforcing cell-cycle activation (62). These findings highlight a recurring theme: circRNA-mediated derepression of cyclin-dependent kinases establishes a permissive proliferative state.

Beyond direct cell-cycle regulators, circRNAs also modulate survival signaling cascades. circRNA-AKT1 sequesters miR-942-5p, resulting in upregulation of AKT1 and activation of the PI3K–AKT pathway (63). AKT signaling promotes cell growth, metabolic adaptation, and resistance to apoptosis. The convergence of circRNA activity on both CDK2-driven cell-cycle machinery and PI3K–AKT–mediated survival underscores the central role of ceRNA networks in sustaining tumor expansion.

In addition to cell-cycle regulation, circRNA-mediated ceRNA networks also enhance tumor cell survival and apoptotic resistance. Activation of the PI3K–AKT pathway represents one of the most important survival-promoting mechanisms in cervical cancer (64, 65). For example, circRNA-AKT1 sequesters miR-942-5p, thereby increasing AKT1 expression and activating downstream survival signaling. Enhanced AKT activity can promote phosphorylation of pro-apoptotic proteins, inhibit caspase-dependent apoptosis, and support metabolic adaptation under cellular stress. This mechanism suggests that circRNAs do not merely accelerate cell-cycle progression but also protect cervical cancer cells from apoptosis, allowing malignant cells to persist during tumor expansion and therapeutic pressure (63). Other circRNA-mediated axes further support this survival-oriented regulatory pattern. Circ_0023404 has been reported to promote malignant progression and chemoresistance by regulating VEGFA and autophagy-related signaling (66). Autophagy may function as a cytoprotective process that enables cervical cancer cells to survive nutrient deprivation, hypoxia, and chemotherapy-induced stress (67, 68). Similarly, circMTO1 has been implicated in cervical cancer tumorigenesis and chemoresistance through miRNA-dependent regulation, suggesting that circRNAs can reinforce anti-apoptotic and stress-adaptive programs beyond direct cell-cycle control (69, 70). These examples indicate that proliferation-related circRNA networks should be viewed as integrated growth–survival modules, in which cell-cycle activation, PI3K–AKT signaling, autophagy, and apoptotic resistance cooperate to sustain cervical cancer progression.

Taken together, proliferation-related ceRNA circuits reveal that circRNAs act upstream of pivotal oncogenic hubs rather than functioning as peripheral regulators. By coordinating transcription factors, cyclin-dependent kinases, PI3K–AKT survival signaling, autophagy-related stress adaptation, and anti-apoptotic pathways, circRNAs integrate cell-cycle acceleration with enhanced tumor cell survival. This dual regulation allows cervical cancer cells not only to proliferate rapidly but also to resist apoptosis and persist under metabolic or therapeutic stress.

### 2.2 Migration, invasion, and EMT-associated circuits

Metastatic dissemination requires coordinated regulation of epithelial–mesenchymal transition (EMT), cytoskeletal remodeling, angiogenesis, extracellular matrix remodeling, and survival adaptation (71–73). However, EMT should be distinguished from general migration or invasion. EMT is a defined cellular reprogramming process in which epithelial tumor cells progressively lose epithelial features, such as E-cadherin-mediated cell–cell adhesion, and acquire mesenchymal characteristics, including increased N-cadherin, Vimentin, Fibronectin, and matrix-remodeling capacity (74, 75). This transition is commonly driven by EMT-associated transcription factors, including SNAIL, SLUG, TWIST, and ZEB1/2, and is functionally associated with enhanced motility, invasiveness, stem-like properties, and therapy resistance (76, 77). Therefore, migration- and angiogenesis-related circRNA axes should be discussed separately from circRNA mechanisms that more directly affect EMT marker shifts or EMT transcriptional programs.

Several circRNA-mediated ceRNA axes mainly promote migration and invasion by regulating angiogenic signaling or cytoskeletal dynamics. Circ_0023404 promotes invasion and metastasis by sponging miR-5047 and enhancing VEGFA expression (66). VEGFA is a critical mediator of angiogenesis, enabling neovascularization that supplies nutrients and oxygen to expanding tumors (78, 79). Increased VEGFA not only supports primary tumor growth but also facilitates metastatic spread by remodeling the tumor microenvironment. In addition, circ_0023404 has been linked to autophagy activation, suggesting that metabolic adaptation and angiogenesis may be co-regulated within this axis (80). Circ0001955 contributes to metastatic progression through the miR-188-3p/NCAPG2 axis (81). NCAPG2 is involved in chromosomal condensation and cell division but has also been associated with enhanced motility and invasive potential (82). By promoting NCAPG2 expression, circ0001955 reinforces both proliferative and migratory phenotypes. Another example, circ_0005576, targets miR-153 to upregulate KIF20A, a kinesin family member implicated in mitotic spindle formation and cell motility (83). Dysregulation of KIF20A enhances cytoskeletal dynamics and facilitates migratory behavior. These examples indicate that some circRNA-mediated ceRNA circuits enhance invasive competence mainly through angiogenesis, cytoskeletal remodeling, and motility-related pathways rather than through direct EMT regulation.

By contrast, circ-HIPK3 provides a more direct example of EMT-associated circRNA regulation in cervical cancer. Circ-HIPK3 promotes EMT by sponging miR-338-3p and upregulating HIF-1α (84). HIF-1α is a hypoxia-responsive transcriptional regulator that can promote EMT-related phenotypic remodeling and enhance metastatic adaptation. Functionally, the circ-HIPK3/miR-338-3p/HIF-1α axis increases cervical cancer cell migration and metastatic potential. Importantly, this mechanism should be interpreted as an EMT-associated ceRNA circuit because it links circRNA activity to HIF-1α-dependent EMT regulation, rather than merely to increased cell motility. Nevertheless, compared with migration-, angiogenesis-, or cytoskeleton-related circRNA axes, direct evidence connecting circRNAs to canonical EMT marker changes, such as E-cadherin loss and N-cadherin or Vimentin induction, remains relatively limited in cervical cancer.

Taken together, current evidence suggests that circRNAs contribute to the acquisition of invasive competence through at least two related but distinct mechanisms. Some circRNAs mainly promote migration and invasion by enhancing angiogenesis, cytoskeletal remodeling, and extracellular matrix-related processes, whereas selected circRNAs, such as circ-HIPK3, are more directly associated with EMT-related regulatory programs. Therefore, circRNAs should be considered contributors to EMT-associated invasive phenotypes in cervical cancer, but broader validation using canonical EMT markers and EMT transcriptional programs is still required before EMT can be defined as a universal central mechanism of circRNA-driven cervical cancer progression.

### 2.3 Chemoresistance-associated ceRNA networks

Therapeutic resistance remains a major obstacle in advanced cervical cancer. Emerging evidence indicates that circRNA-mediated ceRNA networks contribute to chemoresistance by integrating survival signaling, autophagy, and immune modulation. Circ_0023404, beyond its pro-metastatic role, enhances resistance to platinum-based chemotherapy by activating autophagy-related pathways (80, 85, 86). Autophagy can function as a cytoprotective mechanism under therapeutic stress, allowing tumor cells to survive cytotoxic insults. By linking VEGFA upregulation with autophagic adaptation, this circRNA axis connects angiogenesis and survival under treatment pressure. CircMTO1 has also been implicated in promoting tumorigenesis and chemoresistance through miRNA sponging mechanisms, reinforcing proliferative signaling and diminishing apoptotic sensitivity (87). These findings suggest that circRNAs can maintain oncogenic circuits even in the presence of chemotherapeutic agents. More recently, circPRDM4 has been shown to modulate tumorigenesis and immune escape in chemoresistant cervical cancer (88). By influencing immune-related pathways, circPRDM4 bridges classical ceRNA regulation with tumor microenvironment remodeling. This observation is particularly significant, as it indicates that ceRNA networks may extend beyond intrinsic cellular resistance mechanisms to encompass immune-mediated therapeutic evasion.

**Figure 1:**
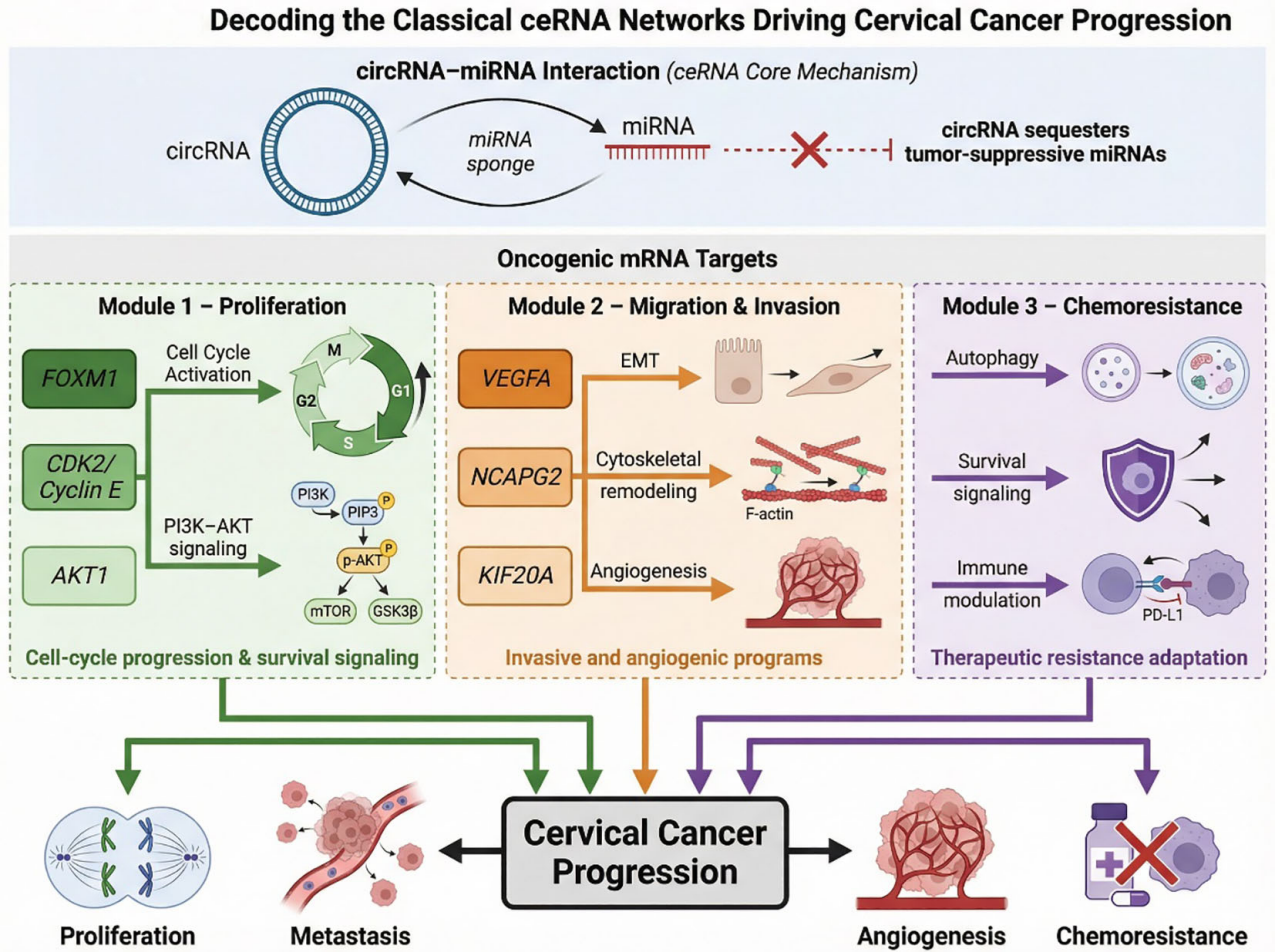
Classical ceRNA networks driving cervical cancer progression. Circular RNAs (circRNAs) function as molecular sponges that sequester tumor-suppressive miRNAs, thereby derepressing oncogenic mRNA targets. These ceRNA interactions converge on key signaling hubs, including cell-cycle regulators (FOXM1, CDK2), PI3K–AKT survival signaling, angiogenic mediators (VEGFA), and EMT-associated factors. Collectively, circRNA-mediated ceRNA circuits promote proliferation, invasion, angiogenesis, and chemoresistance in cervical cancer.

Taken together, classical ceRNA studies establish the first mechanistic layer of circRNA function in cervical cancer. These axes demonstrate that circRNAs promote tumor growth, invasion, angiogenesis, autophagy, and chemoresistance by derepressing oncogenic targets through miRNA sequestration. However, **Table 1** also shows that many ceRNA circuits converge on a limited set of biological programs rather than functioning as fully independent pathways. This convergence provides a rationale for moving from an axis-by-axis description toward a more integrated regulatory framework. In the next section, we discuss how epitranscriptomic modification, RNA-binding protein interactions, and translational control further expand the regulatory capacity of circRNAs in cervical cancer. **Figure 1** showed how classical circRNA–miRNA–mRNA axes converge on core oncogenic pathways that sustain cervical cancer progression (35, 89–91).

Although the ceRNA model has provided a foundational framework for understanding circRNA function in cervical cancer, this paradigm has several inherent limitations. First, many ceRNA interactions are context-dependent and require sufficient abundance of circRNAs, miRNAs, and target mRNAs to exert biologically meaningful effects. Second, the ceRNA model mainly explains post-transcriptional derepression through miRNA sequestration, but it does not fully account for miRNA-independent functions of circRNAs, such as direct interaction with RNA-binding proteins, regulation of RNA stability, modulation of translation, protein scaffolding, and peptide-coding potential (35, 90). Third, ceRNA-based mechanisms alone cannot adequately explain how circRNAs participate in broader tumor biological processes, including epitranscriptomic remodeling, inflammatory signaling, immune escape, metabolic adaptation, and metastatic plasticity (92, 93). Therefore, while classical circRNA–miRNA–mRNA axes remain an important regulatory layer in cervical cancer, they should be viewed as only one component of a more complex circRNA-mediated regulatory network. In the following sections, we further discuss how higher-order mechanisms, including m6A-dependent epitranscriptomic regulation, RNA-binding protein interactions, translational control, and immune-inflammatory signaling, expand the functional landscape of circRNAs beyond the classical ceRNA model.

## 3 Epitranscriptomic regulation and post-transcriptional control by circular RNAs

The ceRNA framework explains how circRNAs regulate oncogenic mRNA expression through miRNA competition, but it does not fully capture mechanisms that directly affect circRNA stability, localization, RNA–protein interactions, or translational output. Recent studies in cervical cancer have identified epitranscriptomic modification and RNA-binding protein-mediated regulation as important mechanisms that shape circRNA function. In this section, we focus on three interconnected layers: m6A-dependent regulation of circRNA stability and activity, non-m6A RNA modifications such as ac4C, and circRNA–RBP interactions that influence mRNA fate and protein synthesis.

### 3.1 m6A-Dependent circRNA regulation

m6A is the most abundant internal modification in eukaryotic mRNA and noncoding RNAs. Its deposition is mediated by methyltransferase “writers” (e.g., METTL3/METTL14), removed by demethylase “erasers” (e.g., ALKBH5), and recognized by “readers” (e.g., YTH domain proteins and IGF2BP family members) (94–96). Increasing evidence indicates that circRNAs are also subject to m6A modification, which influences their stability, localization, and translational potential (97, 98).

In cervical cancer, m6A-dependent circRNA regulation can be understood through a writer–eraser–reader framework. First, m6A “writers” can directly deposit methylation marks on circRNAs and thereby alter their stability and oncogenic output. A recent study showed that METTL3-mediated m6A modification stabilizes circCDK6 and promotes cervical cancer tumorigenesis through the miR-449a/CDK6 axis. Mechanistically, METTL3 directly binds to circCDK6 and installs m6A modification, which enhances circCDK6 stability. Functionally, knockdown of METTL3 or circCDK6 suppresses cervical cancer cell proliferation, induces G0/G1 cell-cycle arrest, and inhibits tumor growth in mouse models. CircCDK6 further acts as a ceRNA to sponge miR-449a, thereby derepressing CDK6 expression and reinforcing cell-cycle progression. This example fills an important mechanistic layer by demonstrating that m6A writers can directly regulate circRNA stability and connect RNA methylation to classical ceRNA-driven oncogenic signaling (99).

Second, m6A “erasers” can remodel circRNA methylation status and influence tumor adaptation programs. ALKBH5-mediated m6A regulation of circCCDC134 represents a critical example. By modulating the m6A status of circCCDC134, ALKBH5 enhances its function and promotes HIF1A transcription, thereby facilitating hypoxia-driven metastatic progression. This mechanism links microenvironmental stress, m6A demethylation, circRNA regulation, and hypoxia-associated tumor plasticity (40).

Third, m6A “readers” recognize methylated circRNAs and determine how m6A marks are translated into biological outputs, including RNA stabilization, translational regulation, RNA turnover, subcellular localization, and therapy-associated phenotypes (43, 44). In cervical cancer, IGF2BP2 provides a representative reader-mediated mechanism. IGF2BP2 binds to m6A-modified circARHGAP12 and enhances its stability, thereby facilitating FOXM1 upregulation and cervical cancer progression (100). This example demonstrates that reader proteins can convert circRNA methylation into sustained oncogenic signaling by protecting m6A-modified circRNAs from degradation. Compared with IGF2BP-mediated stabilization, YTH-domain reader proteins remain less well characterized in cervical cancer circRNA biology and should be interpreted with appropriate caution. YTHDF1 is generally associated with enhanced translation of m6A-modified transcripts, YTHDF2 is frequently linked to RNA decay or turnover, and YTHDF3 can cooperate with YTHDF1 or YTHDF2 to coordinate translation and degradation (40, 101–103). Nuclear YTH-domain proteins, such as YTHDC1, may influence RNA processing, nuclear export, and intracellular localization, whereas YTHDC2 has been implicated in translation and RNA stability control. In the context of circRNAs, these proteins may affect m6A-dependent circRNA stability, cytoplasmic localization, ribosome recruitment, or interaction with downstream effector RNAs and proteins. However, direct evidence defining specific YTH-domain protein–circRNA pairs in cervical cancer remains limited. Therefore, YTH-domain readers should currently be discussed as an emerging regulatory layer that may link m6A-modified circRNAs to translational control, metabolic adaptation, and stress responses, rather than as a fully established mechanism in this disease. In addition, circRNF13 has recently been identified as an m6A-modified circRNA that enhances radioresistance in cervical cancer by increasing CXCL1 mRNA stability (104). Although the precise reader proteins responsible for this effect require further clarification, this finding is clinically relevant because it links m6A-modified circRNA regulation to therapy resistance, inflammatory chemokine signaling, and potential radiosensitization strategies.

Together, these studies indicate that m6A-dependent circRNA regulation in cervical cancer involves coordinated writer, eraser, and reader activities. Writers such as METTL3 can install m6A marks and stabilize oncogenic circRNAs, erasers such as ALKBH5 can remodel circRNA methylation status under hypoxic or metastatic stress, and readers such as IGF2BP2 and potentially YTH-domain proteins can convert m6A marks into downstream functional consequences. Among these layers, YTH-domain reader-mediated regulation remains an important but underexplored area in cervical cancer. Future studies should define specific YTHDF/YTHDC–circRNA interactions and determine whether these readers control circRNA stability, localization, translation, inflammatory signaling, metabolic adaptation, or treatment resistance.

### 3.2 Non-m6A RNA modifications: ac4C and emerging epitranscriptomic layers

Beyond m6A, additional RNA modifications are beginning to emerge as regulators of circRNA-associated networks (105, 106). N4-acetylcytidine (ac4C) is a less extensively studied RNA modification implicated in enhancing mRNA stability and translation efficiency (107, 108). Recent evidence indicates that circMAST1 suppresses cervical cancer progression through modulation of the NAT10/YAP axis. Mechanistically, circMAST1 directly interacts with NAT10, the ac4C “writer” enzyme, and impairs its association with YAP mRNA (107). This interference reduces ac4C modification on YAP transcripts, leading to decreased YAP mRNA stability and subsequent downregulation of YAP-driven oncogenic signaling. By incorporating the upstream enzymatic mechanism, this model provides a more accurate and comprehensive description of how circRNAs can regulate ac4C-dependent RNA modification, linking epitranscriptomic regulation to circRNA-mediated control of tumor-promoting pathways. This finding is significant for two reasons. First, it demonstrates that circRNAs can regulate not only miRNA availability but also RNA modification status of downstream targets. Second, it suggests that circRNAs may function upstream of epitranscriptomic writers or modifiers, thereby shaping RNA fate indirectly. These observations support a broader conceptual shift: circRNAs should not be viewed solely as competitive endogenous RNAs but as regulatory hubs capable of modulating RNA modification landscapes. Through influencing m6A or ac4C-dependent pathways, circRNAs can reshape mRNA stability, translational output, and protein abundance.

### 3.3 RNA-binding proteins and protein–RNA interaction networks

In addition to RNA modification–dependent regulation, circRNAs can interact with specific RNA-binding proteins (RBPs) to modulate mRNA stability, translation, and protein–RNA complex formation (109, 110). In cervical cancer, one experimentally supported example is circ_0005358, which directly interacts with PTBP1 and promotes destabilization of CDCP1 mRNA, thereby suppressing metastatic progression. This mechanism bypasses miRNA-mediated regulation and demonstrates that circRNAs can influence target transcript fate through direct circRNA–RBP interactions (111). m6A-associated RBPs provide another important layer of regulation. For example, IGF2BP2 can recognize m6A-modified circARHGAP12 and enhance its stability, thereby facilitating FOXM1 upregulation and cervical cancer progression (100). YTHDF family proteins, including YTHDF3, have also been implicated in m6A-dependent RNA recognition and translational regulation (103, 112). In this context, m6A-modified circRNAs may recruit or cooperate with reader proteins to influence RNA stability, translation-related processes, or downstream protein output. However, the extent to which specific circRNA–reader complexes directly promote ribosome recruitment in cervical cancer remains to be fully validated. Beyond these experimentally supported examples, circRNAs have been proposed to act as molecular scaffolds that organize RBPs or signaling-associated proteins in other biological contexts. Such scaffold-like functions may help coordinate local RNA metabolism, protein complex assembly, or signaling pathway activation. Nevertheless, for cervical cancer, direct evidence supporting circRNA-mediated assembly of multi-protein signaling complexes remains limited. Therefore, this mechanism should currently be considered an emerging conceptual model rather than a fully established regulatory mode in this disease. Future studies using RNA pull-down, RIP, CLIP-seq, ribosome profiling, polysome fractionation, and proteomics-based interactome mapping will be necessary to define the precise RBPs involved and to determine whether circRNA–RBP complexes directly regulate translation or signaling organization in cervical cancer.

Taken together, these findings redefine circRNAs as active regulators of translational control and protein synthesis rather than passive miRNA sponges. By integrating epitranscriptomic modifications with dynamic RNA-binding protein interactions, circRNAs enhance target mRNA stability, promote translational efficiency, and facilitate increased production of metabolic enzymes and other oncogenic effectors (113–117). Through these multilayered mechanisms, circRNAs amplify signaling cascades and reinforce tumor-promoting pathways, positioning themselves as central modulators of proteomic reprogramming in cervical cancer.

**Figure 2:**
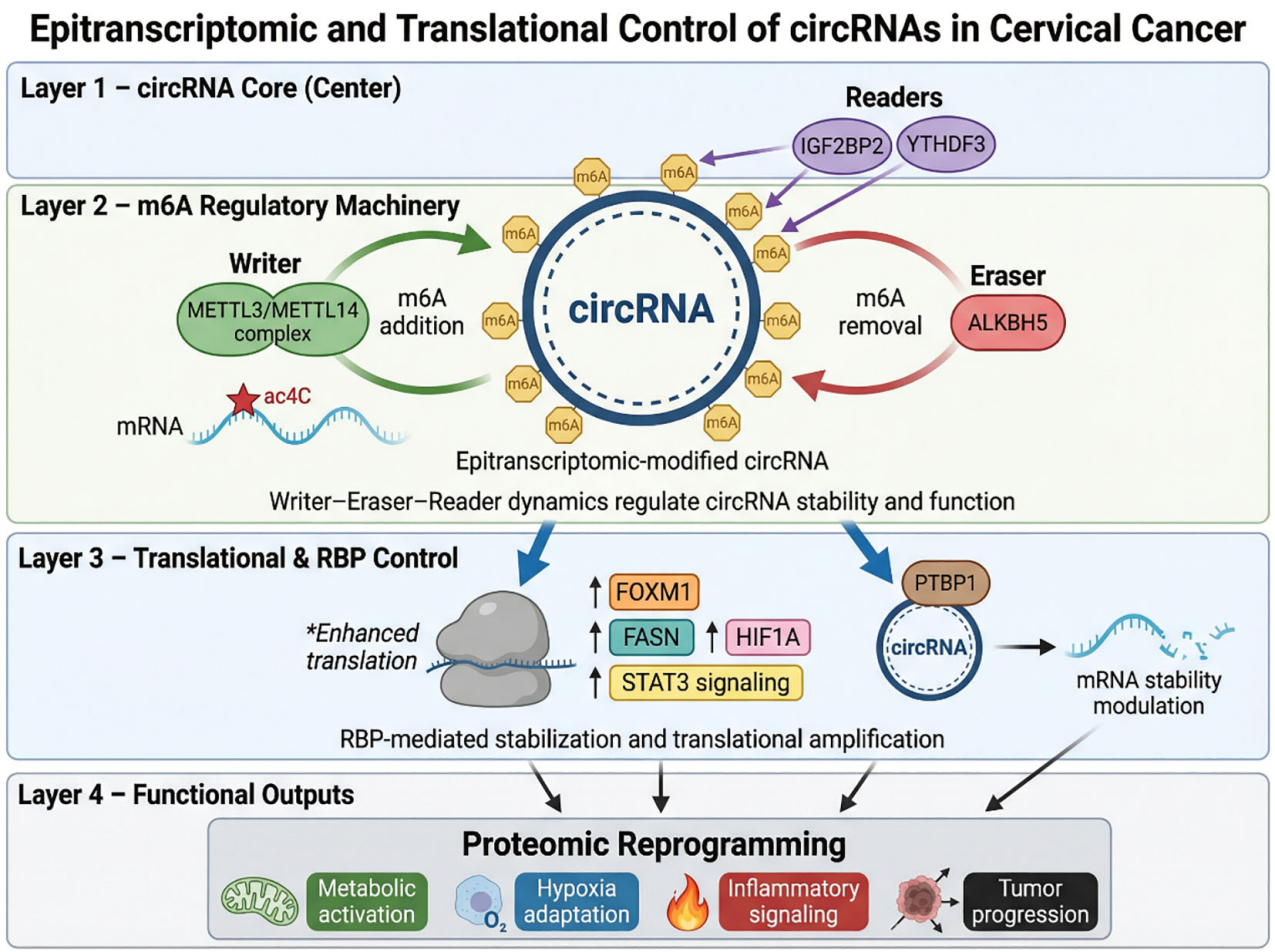
Epitranscriptomic and translational regulation of circRNAs in cervical cancer. m6A and ac4C modifications regulate circRNA stability and function through writer–eraser–reader dynamics. m6A readers such as IGF2BP2 and YTHDF3 bind modified circRNAs, enhancing RNA stability and translational efficiency. circRNA–RBP interactions further promote ribosome recruitment and target mRNA modulation, leading to increased synthesis of oncogenic effectors and amplification of hypoxia, metabolic, and inflammatory signaling pathways.

The emerging evidence from cervical cancer studies indicates that circRNAs occupy a central position at the intersection of epitranscriptomic modification, translational control, and signaling network amplification. The progression from classical ceRNA circuits to m6A- and RBP-mediated regulation reflects a broader paradigm shift in RNA biology. Rather than functioning as isolated post-transcriptional regulators, circRNAs operate as dynamic regulatory nodes that integrate RNA modification machinery, translational control systems, and oncogenic signaling pathways. This expanded functional landscape lays the groundwork for understanding how circRNAs contribute to immune modulation and metastatic reprogramming, which will be discussed in the following section. The epitranscriptomic and RNA-binding protein–mediated regulatory landscape of circRNAs is summarized in **Table 2**. **Figure 2** depicts how epitranscriptomic modifications and RNA-binding protein interactions transform circRNAs into central regulators of translational control. Through stabilizing target transcripts and promoting efficient protein synthesis, circRNAs reinforce oncogenic pathways and reshape the proteomic landscape in cervical cancer.

**Table 2:** Epitranscriptomic and RBP-Mediated Regulation of circRNAs.

circRNA	Regulatory factor	Modification/interaction	Downstream effect	Functional consequence
circCCDC134	ALKBH5	m6A demethylation	↑ HIF1A transcription	Hypoxia adaptation
circARHGAP12	IGF2BP2	m6A reader binding	↑ FOXM1 stability	Proliferation
CircZFR	YTHDF3	m6A reader-mediated translation	↑ FASN translation	Lipid metabolism
circSTX6	m6A machinery	m6A-dependent activation	SPI1–STAT3 activation	Inflammatory signaling
circMAST1	ac4C regulation	Inhibits YAP mRNA ac4C	↓ YAP expression	Tumor suppression
circ_0005358	PTBP1	Protein interaction	↓ CDCP1 stability	Anti-metastatic effect

### 3.4 circRNA-encoded micropeptides: an emerging dimension of translational control

In addition to regulating the translation of linear mRNAs, circRNAs may also function as templates for the direct production of functional micropeptides. Although circRNAs were initially classified as noncoding RNAs, accumulating evidence from multiple tumor types indicates that a subset of circRNAs contains open reading frames and can be translated in a cap-independent manner (118–120). This process is often driven by internal ribosome entry sites, m6A-dependent ribosome recruitment, or other RNA structural elements that enable circRNA translation. CircRNA-derived micropeptides can exert biological functions by modulating signaling pathways, interacting with protein complexes, regulating cellular metabolism, or influencing tumor growth and therapeutic responses (121–123). At present, direct evidence for circRNA-encoded functional micropeptides in cervical cancer remains limited. However, studies in related malignancies have demonstrated that circRNA-derived peptides can act as oncogenic or tumor-suppressive effectors, suggesting that this mechanism may represent an underexplored regulatory layer in cervical cancer. Importantly, m6A modification may facilitate circRNA translation by recruiting reader proteins and translation initiation factors, thereby linking epitranscriptomic regulation to the generation of new protein products (41, 112). This expands the functional significance of circRNAs from indirect post-transcriptional regulators to potential sources of novel effector micropeptides.

Therefore, future studies should systematically evaluate the coding potential of cervical cancer-associated circRNAs using ribosome profiling, mass spectrometry, polysome fractionation, and circRNA-specific overexpression or junction-targeted knockout strategies. Such approaches may help identify previously unrecognized circRNA-derived micropeptides and clarify whether they contribute to HPV-associated oncogenic signaling, immune modulation, metabolic adaptation, metastasis, or therapy resistance in cervical cancer.

## 4 Immune modulation and inflammatory signaling reprogramming

Beyond tumor-intrinsic proliferation and metastatic control, accumulating evidence indicates that circular RNAs (circRNAs) actively participate in immune modulation and inflammatory signaling reprogramming in cervical cancer. Chronic inflammation is a well-established driver of cervical carcinogenesis, particularly in the context of persistent HPV infection. Inflammatory cytokines, transcription factors, and metabolic stress signals collectively shape the tumor microenvironment (TME), promoting immune evasion and malignant progression. Emerging studies suggest that circRNAs are embedded within these regulatory networks, functioning as upstream modulators of cytokine signaling, transcriptional activation, and immune-metabolic adaptation.

### 4.1 IL6/JAK/STAT3 axis

The IL6/JAK/STAT3 pathway represents a central signaling hub linking inflammation, immune suppression, and tumor progression. Persistent activation of STAT3 promotes tumor cell proliferation, angiogenesis, immune tolerance, and resistance to apoptosis, thereby sustaining malignant growth (124). In cervical cancer, emerging evidence connects circRNA regulation to this canonical inflammatory cascade. Notably, m6A-modified circSTX6 has been shown to activate the SPI1–IL6/JAK2/STAT3 signaling axis, reinforcing oncogenic and inflammatory responses. SPI1, a transcription factor involved in immune cell differentiation and cytokine gene regulation, functions upstream to amplify IL6 production and subsequent STAT3 activation (125, 126). Through epitranscriptomic modulation, circSTX6 strengthens this feed-forward signaling loop, thereby accelerating malignant progression.

Beyond tumor-intrinsic proliferation, STAT3 activation exerts profound immunological effects. Hyperactive STAT3 signaling suppresses antitumor immunity by promoting expansion of immunosuppressive cell populations, inducing immune checkpoint molecule expression, inhibiting cytotoxic T-cell activity, and enhancing secretion of anti-inflammatory cytokines (127–131). Consequently, circRNA-mediated reinforcement of the IL6/JAK/STAT3 pathway not only supports tumor survival but also shapes an immunosuppressive microenvironment conducive to immune escape. This mechanism exemplifies how circRNAs integrate epitranscriptomic regulation with inflammatory signal amplification, bridging tumor-intrinsic signaling and immune-extrinsic modulation in cervical cancer.

### 4.2 NF-κB signaling and chronic inflammatory microenvironment

In addition to STAT3, the NF-κB pathway represents another pivotal mediator of inflammation-driven tumor progression in cervical cancer (132–136). NF-κB activation regulates a broad spectrum of genes involved in cytokine production, epithelial–mesenchymal transition (EMT), cell survival, and invasion. Persistent NF-κB signaling sustains a chronic inflammatory microenvironment that facilitates malignant transformation and metastatic dissemination. Notably, circ_0002762 has been shown to promote cervical squamous cell carcinoma progression by activating RelA, a core subunit of the NF-κB complex (137). Through modulation of RelA-dependent transcription, this circRNA enhances inflammatory gene expression and drives invasive phenotypes. NF-κB–dependent transcriptional programs further contribute to upregulation of pro-inflammatory cytokines, induction of EMT, increased matrix metalloproteinase expression, and resistance to apoptosis. Sustained activation of this pathway establishes a feed-forward loop in which inflammation reinforces tumor aggressiveness and metastatic potential. The involvement of circRNAs in NF-κB signaling suggests that they function as modulators of inflammatory tone within the tumor microenvironment, amplifying pro-invasive and pro-survival signals that accelerate cervical cancer progression.

### 4.3 circRNAs in immune escape and tumor microenvironment

**Figure 3:**
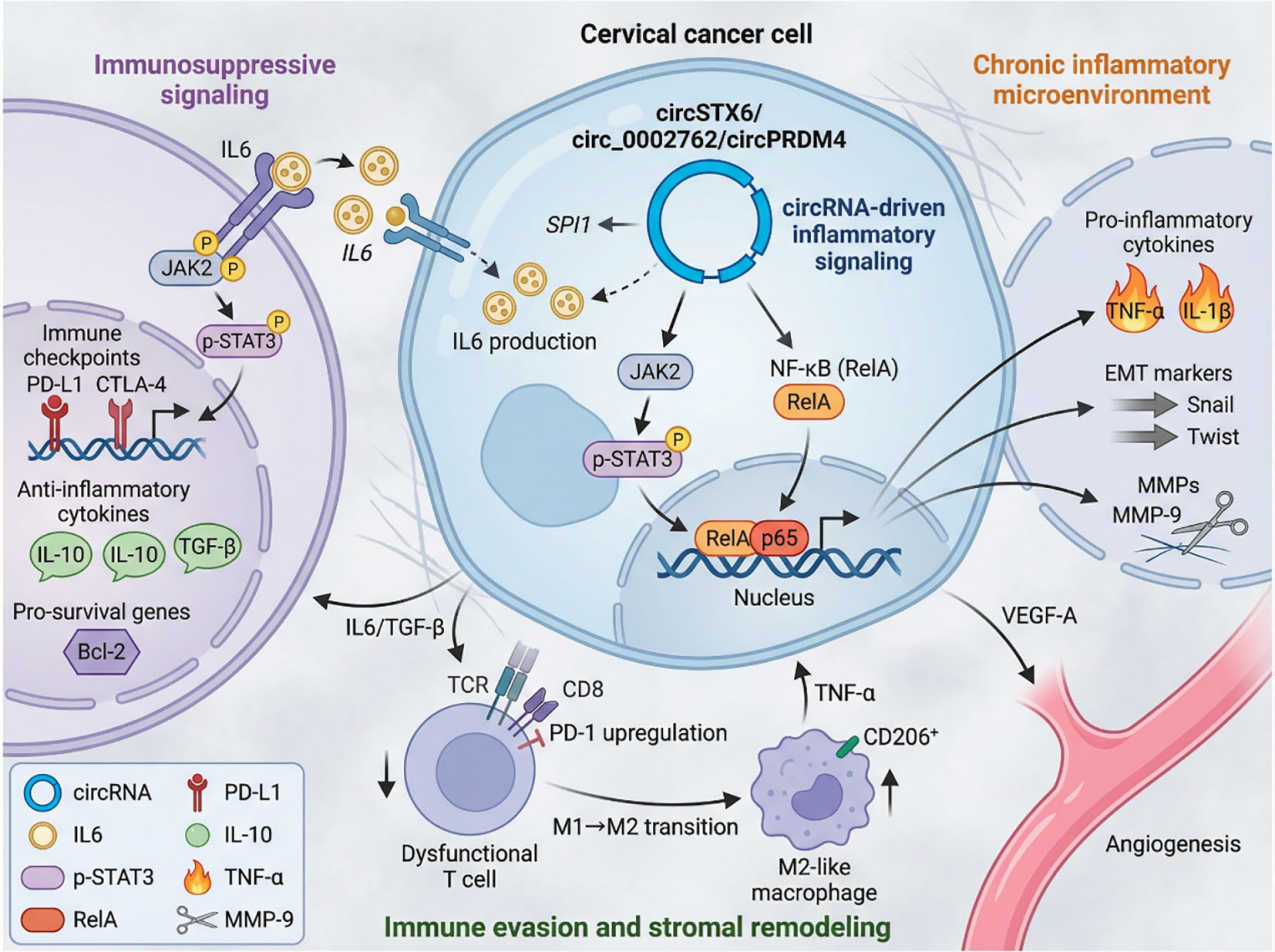
circRNA-mediated immune modulation and inflammatory reprogramming in cervical cancer. circRNAs activate inflammatory pathways, including IL6/JAK/STAT3 and NF-κB signaling, reshaping cytokine networks and transcriptional programs. These pathways suppress antitumor immunity, promote chronic inflammation, and facilitate tumor progression. Through integration of inflammatory signaling and immune modulation, circRNAs contribute to immune escape, metastatic potential, and therapeutic resistance.

Beyond cytokine signaling cascades, circRNAs are increasingly implicated in immune escape and tumor microenvironment (TME) remodeling in cervical cancer. Tumor cells evade immune surveillance by suppressing cytotoxic responses, altering antigen presentation, and reprogramming immune cell populations toward immunosuppressive phenotypes. circPRDM4 has been reported to regulate tumorigenesis and immune escape in chemoresistant cervical cancer (101, 138–140). Although initially characterized within a classical ceRNA framework, its functional impact extends into immune modulation, where it influences immune-related signaling pathways that may reduce immune recognition and enhance tumor survival under therapeutic pressure. These findings suggest that certain circRNAs act as mediators linking intrinsic oncogenic programs with extrinsic immune evasion mechanisms. Moreover, accumulating evidence indicates that circRNAs intersect with metabolic pathways that indirectly shape immune function. Lipid metabolism reprogramming, hypoxia adaptation, and autophagy activation can profoundly influence immune cell infiltration and effector activity within the TME. CircRNA-mediated regulation of metabolic enzymes such as FASN and CD36 may alter nutrient availability and metabolic competition, thereby impairing antitumor immune responses (141–143). Collectively, these observations support a broader conceptual framework in which circRNAs orchestrate an immune–metabolic axis. Through coordinated control of inflammatory signaling, metabolic adaptation, and survival pathways, circRNAs contribute to immune suppression, cytokine network remodeling, enhanced tumor plasticity, and therapeutic resistance in cervical cancer(**Figure 3**).

### 4.4 CircRNA-regulated inflammatory axes and anti-PD-1/PD-L1 immunotherapy response

The inflammatory signaling networks regulated by circRNAs may also influence the response or resistance to anti-PD-1/PD-L1 immunotherapy in cervical cancer (90, 144). Persistent activation of IL6/JAK/STAT3 and NF-κB signaling can reshape the tumor immune microenvironment by promoting immunosuppressive cytokine secretion, reducing cytotoxic T-cell activity, enhancing myeloid-derived suppressor cell and regulatory T-cell accumulation, and inducing immune checkpoint molecule expression (41, 145). In this context, circRNAs that amplify STAT3 or NF-κB activity may indirectly contribute to immune checkpoint blockade resistance by maintaining a chronically inflamed but functionally immunosuppressed tumor niche. For example, m6A-modified circSTX6 activates the SPI1–IL6/JAK2/STAT3 axis, suggesting a potential link between circRNA-dependent epitranscriptomic regulation and immune-suppressive inflammatory signaling (41). STAT3 activation has been associated with impaired antigen presentation, reduced effector T-cell infiltration, increased PD-L1 expression, and resistance to antitumor immunity. Similarly, circ_0002762-mediated activation of RelA/NF-κB signaling may promote secretion of inflammatory cytokines and survival factors that reinforce immune escape and tumor progression (137). Although direct clinical evidence connecting individual circRNAs to anti-PD-1/PD-L1 response in cervical cancer remains limited, these mechanistic findings suggest that circRNA-regulated inflammatory axes may affect the immune contexture that determines checkpoint inhibitor sensitivity.

Therefore, circRNAs involved in IL6/JAK/STAT3, NF-κB, hypoxia, and metabolic reprogramming may serve as candidate biomarkers for immunotherapy stratification. Tumors with circRNA-driven STAT3/NF-κB activation may exhibit an immune-excluded or immune-suppressed phenotype, potentially reducing the efficacy of PD-1/PD-L1 blockade. Conversely, targeting oncogenic circRNAs or their associated inflammatory pathways may help convert immunologically resistant tumors into more responsive states. Future studies should integrate circRNA profiling with PD-L1 expression, tumor mutational burden, HPV antigen presentation, CD8+ T-cell infiltration, myeloid signatures, and clinical outcomes after immune checkpoint blockade to clarify the predictive and therapeutic relevance of circRNA-regulated inflammatory networks in cervical cancer.

## 5 Metastatic reprogramming and tumor plasticity

Metastasis is not merely a consequence of increased cellular motility but a highly coordinated evolutionary process involving metabolic adaptation, microenvironmental remodeling, and survival reprogramming. Tumor plasticity—the ability of cancer cells to dynamically adjust their phenotype in response to environmental stress—is central to metastatic dissemination and colonization. Emerging evidence indicates that circRNAs contribute to this adaptive landscape by orchestrating metabolic rewiring, hypoxia signaling, lymphangiogenesis, and stress-response pathways. These functions position circRNAs as key regulators of metastatic reprogramming in cervical cancer.

### 5.1 Lipid metabolism and metastatic energy supply

Metastatic progression requires substantial bioenergetic and biosynthetic resources. Lipid metabolism has emerged as a critical driver of tumor invasion and dissemination, providing membrane components, signaling lipids, and energy substrates. CircZFR has been shown to promote lymph node metastasis by enhancing translation of fatty acid synthase (FASN) through m6A reader–dependent mechanisms (112). FASN is a key enzyme responsible for *de novo* lipogenesis, enabling tumor cells to synthesize fatty acids required for membrane expansion and signaling molecule production. Elevated FASN activity not only fuels rapid proliferation but also enhances migratory capacity and resistance to oxidative stress. By facilitating FASN translation, CircZFR directly links epitranscriptomic regulation to metabolic reprogramming and metastatic competence. Similarly, circ_ASH1L promotes malignant progression by targeting CD36, a lipid uptake receptor involved in fatty acid transport (146). CD36-mediated lipid acquisition supports energy production and contributes to metastatic niche adaptation. Increased lipid uptake can also influence redox balance and promote survival under detachment or hypoxic stress. Together, these findings suggest that circRNAs regulate both endogenous lipid synthesis and exogenous lipid uptake, thereby optimizing metabolic flexibility required for metastatic dissemination.

### 5.2 Hypoxia signaling and adaptive plasticity

Hypoxia is a defining feature of solid tumors and a major driver of metastasis. Hypoxia-inducible factor 1α (HIF1A) orchestrates transcriptional programs that promote angiogenesis, glycolysis, EMT, and survival under low oxygen conditions (147–150). CircCCDC134 has been reported to enhance HIF1A transcription through epitranscriptomic regulation mediated by ALKBH5. By amplifying HIF1A signaling, circCCDC134 promotes hypoxia adaptation and metastatic progression. Hypoxia-induced gene expression reprograms cellular metabolism toward glycolysis, enhances VEGF-mediated angiogenesis, and facilitates EMT, collectively increasing invasive potential. Importantly, hypoxia also contributes to immune suppression and therapeutic resistance. Therefore, circRNA-mediated activation of HIF1A not only enhances metastatic plasticity but may also reshape the tumor microenvironment in favor of tumor persistence.

### 5.3 Lymphangiogenesis and metastatic niche formation

Lymphatic dissemination is a common route of metastasis in cervical cancer. Successful metastatic colonization requires formation of a supportive pre-metastatic niche characterized by enhanced lymphangiogenesis and stromal remodeling. LNMAC has been shown to promote lymphatic metastasis through epigenetic regulation of FGF2-induced lymphangiogenesis (151). FGF2 is a potent pro-angiogenic and pro-lymphangiogenic factor that stimulates endothelial cell proliferation and vessel formation (152–154). By enhancing FGF2-driven signaling, LNMAC contributes to expansion of lymphatic networks that facilitate tumor cell dissemination. This mechanism underscores an important concept: circRNAs not only regulate tumor cell–intrinsic pathways but also modulate stromal and vascular components of the microenvironment. Through promoting lymphangiogenesis, circRNAs actively shape metastatic niches that support tumor spread.

### 5.4 Autophagy and survival adaptation

Metastatic cells encounter multiple stressors, including detachment from the extracellular matrix, nutrient deprivation, oxidative stress, and chemotherapeutic exposure. Autophagy serves as a cytoprotective mechanism that enables tumor cells to survive under such adverse conditions (155–159). Circ_0023404 has been implicated in activating autophagy signaling while simultaneously enhancing VEGFA expression. This dual function integrates survival adaptation with angiogenic remodeling. By promoting autophagy, circ_0023404 may help metastatic cells withstand metabolic stress and therapeutic pressure (66). Moreover, autophagy can modulate immune responses and influence antigen presentation, further contributing to tumor persistence. Through coordinating angiogenesis, autophagy, and metabolic adaptation, circ_0023404 exemplifies how circRNAs integrate multiple survival pathways to enhance metastatic resilience.

**Figure 4:**
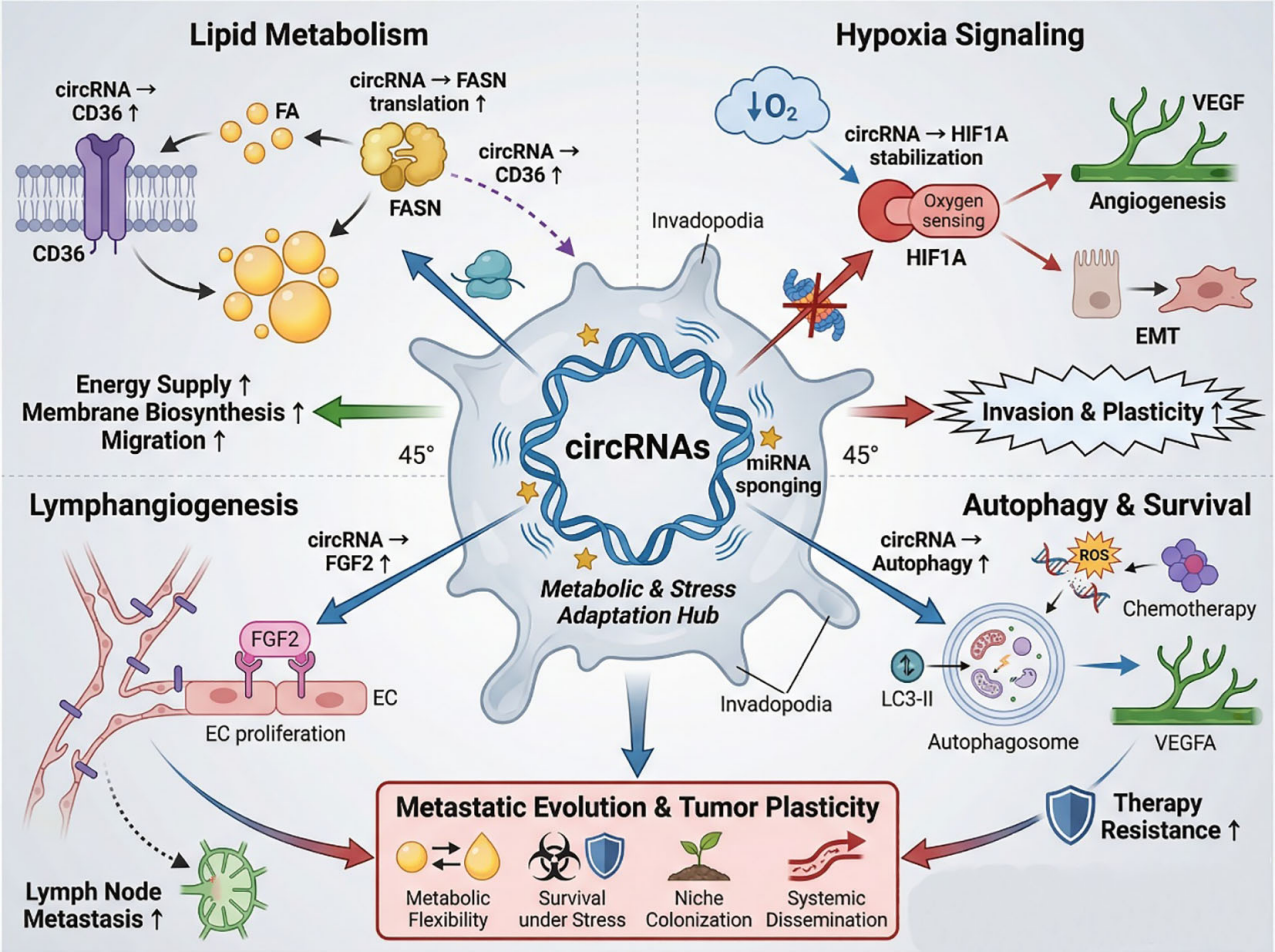
CircRNA-driven metastatic reprogramming and tumor plasticity in cervical cancer. CircRNAs coordinate lipid metabolism (FASN, CD36), hypoxia signaling (HIF1A), lymphangiogenesis (FGF2), and autophagy activation to promote metabolic flexibility and stress adaptation. Through integrating metabolic rewiring and microenvironmental remodeling, circRNAs enhance metastatic competence, niche formation, and therapeutic resistance in cervical cancer.

Collectively, these findings suggest that circRNAs regulate metastatic progression through multilayered mechanisms involving lipid metabolism, hypoxia adaptation, lymphangiogenesis, and stress-response pathways. Rather than acting as isolated regulators, circRNAs integrate epitranscriptomic modification, metabolic rewiring, and microenvironmental remodeling to promote tumor plasticity. By enhancing energy supply, facilitating niche formation, and enabling survival under stress, circRNAs contribute to the dynamic evolution of cervical cancer from localized disease to systemic metastasis. This metastatic reprogramming represents the culmination of circRNA-mediated regulatory networks spanning ceRNA control, epitranscriptomic modification, immune modulation, and metabolic adaptation(**Figure 4**). circRNA-driven immune and metabolic reprogramming mechanisms contributing to metastatic evolution are summarized in **Table 3**.

**Table 3:** circRNA-mediated immune and metabolic reprogramming in cervical cancer.

circRNA	Pathway/target	Immune or metabolic role	Impact on tumor progression
circSTX6	IL6/JAK/STAT3	Immune suppression	Tumor growth & immune escape
circ_0002762	NF-κB (RelA)	Chronic inflammation	Invasion & metastasis
circPRDM4	Immune pathways	Immune evasion	Chemoresistance
CircZFR	FASN	Lipid synthesis	Lymph node metastasis
circ_ASH1L	CD36	Lipid uptake	Metabolic adaptation
circCCDC134	HIF1A	Hypoxia signaling	Metastatic plasticity
LNMAC	FGF2	Lymphangiogenesis	Metastatic niche formation
circ_0023404	Autophagy + VEGFA	Survival adaptation	Chemoresistance & invasion

## 6 Integrated regulatory landscape: a unified model

The accumulating evidence discussed above reveals that circRNAs in cervical cancer operate within a multilayered regulatory architecture rather than through isolated molecular axes. Classical ceRNA circuits represent the foundational layer, where circRNAs modulate oncogene expression by sequestering tumor-suppressive microRNAs. However, emerging data clearly demonstrate that this represents only the initial tier of circRNA-mediated regulation. A more comprehensive model must incorporate epitranscriptomic modification, RNA-binding protein (RBP) interactions, inflammatory signaling, metabolic reprogramming, and metastatic adaptation into a unified conceptual framework. At the first regulatory layer, circRNAs function as ceRNAs to derepress key oncogenic drivers such as FOXM1, CDK2, AKT1, VEGFA, and KIF20A (160–164). These interactions establish proliferative and invasive competence. The second layer involves epitranscriptomic control, particularly m6A and ac4C modifications, which influence circRNA stability, localization, and translational potential. Through writer–eraser–reader dynamics, circRNAs are stabilized or activated, thereby enhancing their regulatory output. Importantly, m6A readers such as YTHDF3 and IGF2BP2 integrate circRNAs directly into the translational machinery, linking RNA modification to increased protein synthesis and metabolic enzyme production.

The third regulatory tier centers on RBP-mediated scaffolding and translational control. By interacting with proteins such as PTBP1, circRNAs modulate mRNA stability and promote formation of functional protein complexes (165–167). This layer reinforces oncogenic signaling pathways at the post-transcriptional and translational levels. Beyond tumor-intrinsic processes, circRNAs extend their influence to inflammatory and immune pathways, including IL6/JAK/STAT3 and NF-κB signaling. Through these mechanisms, circRNAs reshape cytokine networks, promote immune suppression, and amplify chronic inflammatory signaling within the tumor microenvironment. At the highest level of integration, circRNAs contribute to metabolic reprogramming and metastatic evolution. By enhancing lipid synthesis (FASN), lipid uptake (CD36), hypoxia adaptation (HIF1A), lymphangiogenesis (FGF2), and autophagy activation, circRNAs coordinate the energetic and microenvironmental adaptations necessary for metastatic dissemination (168). Thus, circRNAs bridge molecular signaling, metabolic flexibility, immune evasion, and niche formation, driving tumor plasticity and systemic progression.

Taken together, we propose a unified model in which circRNAs act as central regulatory nodes that connect: ceRNA derepression → epitranscriptomic modulation → RBP-dependent translational control → inflammatory amplification → metabolic rewiring → metastatic reprogramming.

Rather than functioning in linear pathways, these layers form an interconnected regulatory network with multiple feedback loops. Epitranscriptomic modifications can enhance circRNA stability, which strengthens ceRNA activity; inflammatory signaling can promote metabolic adaptation; metabolic stress can further reshape immune responses. CircRNAs, therefore, occupy a strategic position within this dynamic circuitry, enabling coordinated control over tumor evolution. This integrative perspective highlights circRNAs as master regulators of cervical cancer progression and provides a conceptual foundation for therapeutic intervention. Targeting circRNAs or their associated modification and translation machinery may simultaneously disrupt oncogenic signaling, immune suppression, and metastatic plasticity. To improve circRNA-to-function specificity and avoid repetitive interpretation of recurrent examples, representative circRNAs were further mapped according to their dominant biological functions (**Table 4**). Some circRNAs primarily illustrate a single regulatory program, such as circCLK3 and circ_0000520 in proliferation-related ceRNA networks. Others, including circ_0023404, circCCDC134, circRNF13, and circZFR, are discussed across more than one section because they connect multiple biological processes, such as angiogenesis, autophagy, hypoxia adaptation, lipid metabolism, or therapy resistance. Therefore, these circRNAs are interpreted as multifunctional nodes rather than repeatedly used isolated examples.

**Table 4:** Thematic organization of circRNA-targeted therapeutic strategies in cervical cancer.

Therapeutic theme	Representative circRNA axes	Biological rationale	Potential strategy
Proliferation and survival	circCLK3/FOXM1; circ_0000520/CDK2; circCDK6/CDK6; circRNA-AKT1/AKT1	Cell-cycle progression and survival signaling	Junction-targeted siRNA/ASO; combination with chemotherapy
Hypoxia and metastatic plasticity	circCCDC134/HIF1A; circ-HIPK3/HIF-1α; LNMAC/FGF2	Hypoxia adaptation, EMT-associated invasion, lymphangiogenesis	circRNA silencing; anti-metastatic combination therapy
Inflammation and immune suppression	circSTX6/IL6-JAK-STAT3; circ_0002762/RelA-NF-κB; circPRDM4	Cytokine signaling, immune escape, inflammatory TME	Combine circRNA targeting with immunotherapy
Therapy resistance and stress adaptation	circ_0023404/VEGFA-autophagy; circRNF13/CXCL1	Chemoresistance, radioresistance, autophagy, inflammatory chemokine signaling	Radiosensitization or chemosensitization strategies

## 7 Clinical implications and therapeutic opportunities

The expanding understanding of circRNA-mediated regulatory networks in cervical cancer not only advances mechanistic insight but also opens new avenues for clinical translation. Given their exceptional stability, tissue specificity, and regulatory versatility, circRNAs hold considerable promise as diagnostic biomarkers, prognostic indicators, and therapeutic targets. Integrating circRNA biology into precision oncology frameworks may facilitate improved patient stratification and the development of novel treatment strategies.

### 7.1 circRNAs as diagnostic and prognostic biomarkers

One of the most attractive features of circRNAs is their covalently closed structure, which confers resistance to exonuclease degradation and ensures remarkable stability in tissues, plasma, serum, and other bodily fluids (119, 169, 170). This stability makes circRNAs particularly suitable for liquid biopsy applications. In cervical cancer, several dysregulated circRNAs have been associated with tumor proliferation, invasion, metastasis, and therapy resistance, suggesting their potential value as diagnostic or prognostic biomarkers.

Representative examples include circCLK3, which promotes cervical cancer progression through the miR-320a/FOXM1 axis, and circ_0000520, which enhances cell proliferation by regulating the miR-1296/CDK2 pathway (60, 61). CircZFR has also been implicated in cell-cycle progression through regulation of the SSBP1/CDK2/cyclin E1 complex (62). These proliferation-associated circRNAs may reflect tumor growth activity and could potentially contribute to risk stratification. In addition, circ_0023404 is linked to cervical cancer metastasis and chemoresistance through VEGFA and autophagy-related signaling, indicating that specific circRNA expression patterns may correlate with aggressive tumor behavior and therapeutic failure (66). CircRNAs involved in higher-order regulatory mechanisms may provide additional biomarker value. For example, circCCDC134 is associated with ALKBH5-mediated m6A regulation and HIF1A-driven hypoxia adaptation, suggesting potential relevance to metastatic progression (40). m6A-modified circSTX6 activates the SPI1–IL6/JAK2/STAT3 axis, linking circRNA dysregulation with inflammatory signaling and immune modulation (41). Moreover, circRNF13 has been associated with radioresistance through CXCL1 mRNA stabilization, providing a clinically relevant example of a circRNA-related marker connected to treatment response (104). Therefore, circRNA signatures may be particularly informative when integrated with pathway activity, lymph node status, hypoxia markers, inflammatory signaling, immune contexture, and therapeutic outcomes.

Importantly, circRNA-based biomarkers may outperform single-gene markers by capturing multilayered regulatory states, including ceRNA activity, epitranscriptomic regulation, immune modulation, metabolic adaptation, and treatment resistance. However, most candidate circRNA biomarkers in cervical cancer remain at the discovery or preclinical validation stage. Large independent cohorts, standardized detection methods, longitudinal liquid biopsy studies, and integration with clinicopathological parameters will be required before circRNA signatures can be translated into routine diagnostic or prognostic tools.

### 7.2 Exosomal circRNAs and intercellular communication

CircRNAs are not restricted to intracellular compartments but can be selectively packaged into extracellular vesicles, particularly exosomes, and released into circulation (31, 171). Owing to their covalently closed structure and high stability, exosomal circRNAs can persist in blood and other body fluids, making them attractive candidates for noninvasive biomarker development and dynamic monitoring of tumor progression. Functionally, exosomal circRNAs may mediate intercellular communication within the tumor microenvironment by transferring regulatory RNA signals from tumor cells to stromal cells, immune cells, endothelial cells, or distant recipient cells (172, 173).

In cervical cancer, direct evidence for exosomal circRNA-mediated communication remains relatively limited compared with some other malignancies. Nevertheless, several cervical cancer-associated circRNAs provide biologically plausible candidates for future investigation because they are linked to processes that require tumor–microenvironment crosstalk. For example, circ_0023404 regulates VEGFA and autophagy-related signaling, suggesting potential relevance to angiogenesis, stress adaptation, and chemoresistance (66). LNMAC promotes FGF2-associated lymphangiogenesis and lymphatic metastasis, indicating a possible connection between circRNA regulation and vascular niche remodeling (151). CircPRDM4 has been associated with chemoresistant cervical cancer and immune escape, making it a candidate for exploring whether circRNA-containing extracellular vesicles participate in transmission of therapy-resistant or immunosuppressive phenotypes (88).

If these or related circRNAs are selectively enriched in tumor-derived exosomes, they may contribute to angiogenic remodeling, lymphangiogenesis, metastatic niche formation, immune suppression, or intercellular transfer of treatment resistance. From a clinical perspective, exosomal circRNAs may be particularly useful for liquid biopsy because they could reflect both tumor-intrinsic molecular states and tumor–microenvironment interactions. Future studies should therefore determine whether cervical cancer-associated circRNAs such as circ_0023404, LNMAC, circPRDM4, circCCDC134, circSTX6, or circRNF13 are detectable in plasma-derived exosomes and whether their abundance correlates with lymph node metastasis, recurrence, radiotherapy or chemotherapy resistance, PD-1/PD-L1 blockade response, or patient prognosis.

Therapeutically, disrupting exosome-mediated circRNA transfer may represent a potential strategy to interfere with tumor–microenvironment communication. However, this concept remains largely exploratory in cervical cancer. Further studies using patient-derived exosomes, exosome-tracing assays, organoid–immune co-culture systems, and *in vivo* metastasis models are needed to validate whether specific exosomal circRNAs functionally drive angiogenesis, immune modulation, metastatic niche formation, or therapy resistance.

### 7.3Therapeutic targeting of circRNA-driven biological programs

Given their oncogenic roles, direct targeting of circRNAs represents a logical therapeutic approach. Unlike linear transcripts, circRNAs contain unique back-splice junction sequences that allow highly specific targeting of the circular transcript while minimizing effects on the corresponding linear host gene (118, 119). Small interfering RNAs (siRNAs) or antisense oligonucleotides (ASOs) designed against back-splice junctions can selectively degrade oncogenic circRNAs or block their interactions with miRNAs, RNA-binding proteins, or downstream effector molecules (120, 174). Rather than considering each circRNA as an isolated therapeutic target, circRNA-directed strategies may be more coherently organized according to the biological programs they regulate.

First, circRNAs that reinforce cell-cycle progression and proliferative survival may represent targets for growth-suppressive intervention. This group includes circCLK3, which regulates the miR-320a/FOXM1 axis, circ_0000520, which promotes the miR-1296/CDK2 pathway, circCDK6, which acts through the miR-449a/CDK6 axis, and circRNA-AKT1, which enhances miR-942-5p/AKT1-dependent survival signaling (60, 61, 63, 99). Therapeutic silencing of these circRNAs may attenuate FOXM1-, CDK2/CDK6-, or AKT-associated proliferative programs and potentially increase tumor cell vulnerability to cytotoxic treatment.

Second, circRNAs involved in hypoxia adaptation and metastatic plasticity may provide opportunities to interfere with tumor dissemination. CircCCDC134 links ALKBH5-dependent m6A regulation to HIF1A transcription and hypoxia-associated metastatic progression (40). Circ-HIPK3 promotes an EMT-associated invasive phenotype through the miR-338-3p/HIF-1α axis (84). In addition, circRNAs associated with lipid metabolism, lymphangiogenesis, or metastatic niche formation, such as circZFR/FASN, circ_ASH1L/CD36, and LNMAC/FGF2-related signaling, may represent candidates for blocking metabolic flexibility and lymphatic spread (112, 146, 151). These targets are particularly relevant for advanced or metastatic cervical cancer, where hypoxia, metabolic adaptation, and lymphatic dissemination contribute to poor outcome.

Third, circRNA-regulated inflammatory and immune-suppressive pathways may be considered as immunomodulatory targets. For example, m6A-modified circSTX6 activates the SPI1–IL6/JAK2/STAT3 axis, whereas circ_0002762 activates RelA/NF-κB signaling (41, 137). Because STAT3 and NF-κB pathways can promote inflammatory cytokine production, immune suppression, survival signaling, and potentially immune checkpoint resistance, targeting these circRNA-associated inflammatory circuits may help remodel an immunosuppressive tumor microenvironment. CircPRDM4, which has been associated with chemoresistant cervical cancer and immune escape, may also provide a rationale for combining circRNA-directed therapy with chemotherapy or immunotherapy (88, 175).

Fourth, circRNAs that support therapy resistance and stress adaptation may serve as sensitizing targets. Circ_0023404 connects VEGFA-associated angiogenic signaling with autophagy-related stress adaptation and chemoresistance (66). CircRNF13 has been implicated in radioresistance through CXCL1 mRNA stabilization, suggesting that its inhibition may enhance radiosensitivity (104). In this context, circRNA-targeted siRNA or ASO strategies may not necessarily function as standalone therapies but may be more useful as combination approaches with platinum-based chemotherapy, radiotherapy, anti-angiogenic therapy, or immune checkpoint blockade.

Despite these opportunities, circRNA-targeted therapy in cervical cancer remains at an early preclinical stage. Major challenges include efficient delivery to tumor tissues, avoidance of off-target effects, sufficient intracellular uptake, immune activation by nucleic acid therapeutics, and validation of target specificity at the back-splice junction. Nanoparticle-based delivery platforms, ligand-conjugated oligonucleotides, and exosome-mimetic carriers are being explored to improve RNA therapeutic stability and tumor selectivity. Future studies using patient-derived organoids, xenograft models, immune-competent models, and rational combination treatment designs will be necessary to determine whether circRNA-targeted siRNA or ASO strategies can be translated into clinically feasible therapies for cervical cancer.

### 7.4 Targeting epitranscriptomic machinery

Because several oncogenic circRNAs in cervical cancer are regulated by RNA modifications, targeting epitranscriptomic machinery represents an indirect but potentially valuable therapeutic strategy. Rather than viewing m6A regulators as nonspecific global modifiers, future therapeutic development should be guided by defined m6A regulator–circRNA–pathway axes (40, 112, 176). In this context, m6A “writers,” “erasers,” and “readers” may influence circRNA stability, localization, RNA-binding protein recognition, translational potential, and downstream oncogenic signaling.

For m6A writers, METTL3-mediated stabilization of circCDK6 provides a representative example linking RNA methylation to cell-cycle progression. METTL3 can promote circCDK6 stability, while circCDK6 acts through the miR-449a/CDK6 axis to enhance cervical cancer cell proliferation. This suggests that disrupting METTL3-dependent circRNA stabilization may attenuate CDK6-driven proliferative signaling (99). For m6A erasers, ALKBH5-mediated regulation of circCCDC134 connects demethylation activity with HIF1A transcription, hypoxia adaptation, and metastatic progression. Inhibition of this axis could potentially weaken hypoxia-driven tumor plasticity and metastatic competence (40). For m6A readers, IGF2BP2-dependent stabilization of m6A-modified circARHGAP12 supports FOXM1 upregulation, whereas YTHDF-related mechanisms may connect m6A-modified circRNAs with translational and metabolic regulation (100). Epitranscriptomic targeting may also have implications for treatment resistance and immune-inflammatory signaling. CircRNF13 has been associated with cervical cancer radioresistance through CXCL1 mRNA stabilization, providing a clinically relevant example linking m6A-modified circRNA regulation to therapeutic response (104). In addition, m6A-modified circSTX6 activates the SPI1–IL6/JAK2/STAT3 axis, suggesting that m6A-regulated circRNAs may contribute to inflammatory signaling, immune escape, and potentially resistance to immunotherapy (41). Therefore, targeting epitranscriptomic regulators may be most effective when combined with pathway-level biomarkers, such as HIF1A activation, FOXM1 expression, CXCL1 signaling, STAT3 activation, or immune checkpoint status. However, several challenges should be considered. m6A regulators have broad effects on both coding and noncoding RNAs, and systemic inhibition may cause unintended transcriptome-wide consequences. Therefore, therapeutic strategies should aim to identify tumor-selective dependencies, context-specific circRNA targets, and combination regimens rather than broadly suppressing m6A machinery. Future studies should evaluate whether inhibition of METTL3-, ALKBH5-, IGF2BP2-, or YTHDF-dependent circRNA axes can enhance sensitivity to chemotherapy, radiotherapy, anti-angiogenic therapy, or anti-PD-1/PD-L1 immunotherapy in cervical cancer models.

## 8 Future perspectives: key gaps and future directions

Several key gaps must be addressed before circRNA biology can be translated into a more precise understanding of cervical cancer progression and treatment response. First, the cellular and spatial specificity of cervical cancer-associated circRNAs remains insufficiently defined. Most available studies are based on bulk tumor samples or cell-line models, which cannot determine whether specific circRNAs are enriched in malignant epithelial cells, stromal cells, endothelial cells, immune subsets, hypoxic niches, invasive fronts, or lymphovascular regions (118, 177, 178). Future studies should integrate improved circRNA-detection algorithms with single-cell RNA sequencing, long-read sequencing, spatial transcriptomics, and imaging-based validation to define cell-type- and niche-specific circRNA programs.

Second, the functional hierarchy of circRNA mechanisms remains unclear. Many cervical cancer studies identify circRNA–miRNA–mRNA axes, but fewer studies directly compare ceRNA activity with m6A-dependent regulation, RBP-mediated RNA control, exosomal transfer, or peptide-coding potential within the same experimental system. Future work should prioritize mechanistic validation using junction-specific knockdown or knockout, rescue experiments, RNA pull-down, RIP/CLIP-based interaction mapping, ribosome profiling, polysome fractionation, and mass spectrometry. These approaches will help distinguish validated mechanisms from predicted regulatory networks.

Third, the relationship between circRNAs and the immune microenvironment remains underexplored. Although several circRNAs have been linked to inflammatory pathways, direct evidence connecting specific circRNAs to immune-cell infiltration, antigen presentation, CD8+ T-cell dysfunction, macrophage polarization, regulatory T-cell accumulation, or response to PD-1/PD-L1 blockade remains limited (90, 144). Future studies should combine circRNA profiling with immune phenotyping, cytokine analysis, HPV antigen-presentation assessment, and clinical immunotherapy outcomes.

Fourth, the clinical value of circRNAs as biomarkers requires more rigorous validation. Most candidate circRNAs have been identified in small retrospective cohorts or preclinical models. Large multicenter cohorts, standardized detection platforms, longitudinal liquid-biopsy designs, and integration with clinicopathological variables are needed to determine whether circRNA signatures can improve diagnosis, prognostic stratification, metastasis prediction, or treatment-response monitoring.

Finally, circRNA-targeted therapy remains at an early stage. Future translational studies should address delivery efficiency, tumor specificity, off-target effects, immune activation by nucleic acid therapeutics, and the feasibility of combining circRNA-targeted siRNA or ASO strategies with chemotherapy, radiotherapy, anti-angiogenic therapy, or immunotherapy. These studies should be performed in patient-derived organoids, immune-competent models, and clinically relevant metastasis or therapy-resistance models.

## 9 Conclusion

CircRNAs are important post-transcriptional regulators in cervical cancer and participate in tumor progression through ceRNA-dependent and ceRNA-independent mechanisms. Current evidence supports their involvement in oncogenic signaling, RNA modification-associated regulation, inflammatory remodeling, metabolic adaptation, metastasis, and treatment resistance. However, many proposed mechanisms remain incompletely validated, particularly those related to RBP-mediated translation, immune-checkpoint response, exosomal communication, and circRNA-derived peptides. Therefore, circRNAs should be viewed as promising but still emerging biomarkers and therapeutic targets. Future progress will depend on rigorous functional validation, evidence-based mechanistic classification, and clinically relevant models that connect circRNA biology with patient stratification and therapeutic response. In summary, the distinct contribution of this review is not the re-listing of circRNA–miRNA–mRNA axes that have been summarized in previous articles, but the construction of an integrated regulatory model in which circRNAs connect ceRNA activity with epitranscriptomic modification, RBP-mediated translational control, immune-inflammatory remodeling, metabolic adaptation, and metastatic plasticity. This framework may help prioritize future studies that move from descriptive circRNA profiling toward mechanism-based validation, spatial and single-cell mapping, biomarker stratification, and rational therapeutic targeting.
